# Burden of multimorbidity and verbal phonemic fluency in cognitively healthy and mildly impaired older adults: findings from a real-world study

**DOI:** 10.1007/s40520-025-03133-1

**Published:** 2025-10-08

**Authors:** Elisa Fabbri, Virginia Boccardi, Anna Giulia Guazzarini, Ilenia Murasecco, Francesco Melis, Patrizia Bastiani, Paolo Muratori, Carmelinda Ruggiero, Patrizia Mecocci

**Affiliations:** 1 Department of Medical and Surgical Sciences, University of Bologna, Bologna, Italy; 2 Division of Internal Medicine, Morgagni- Pierantoni Hospital, Forlì, Italy; 3 Division of Gerontology and Geriatrics, Department of Medicine and Surgery, University of Perugia, Perugia, Italy; 4 Department for Life Quality Studies, University of Bologna, Bologna, Italy; 5 Division of Clinical Geriatrics, Department of Neurobiology, Care Sciences and Society, Karolinska Institutet, Stockholm, Sweden

**Keywords:** Aging, Cognition, Multimorbidity, Verbal fluency

## Abstract

**Objective:**

To examine the association between burden of multimorbidity and cognitive function in older adults with normal cognition or mild cognitive impairment (MCI).

**Methods:**

Data from electronic health records of 898 individuals cognitively healthy or with MCI were included. Burden of multimorbidity was assessed using Cumulative Illness Rating Scale-Geriatrics (CIRS-G) total score, while cognitive function was evaluated using a comprehensive battery of neuropsychological tests. Age, sex, education, basic activities of daily living and instrumental activities of daily living scores, and total number of current medications were covariates. Spearmen’s correlations and multivariate regression models investigated the cross-sectional association between burden of multimorbidity and cognitive function.

**Results:**

At a first exploratory analysis, higher CIRS-G score was significantly and negatively correlated with Addenbrooke’s Cognitive Examination Revised (ACE-R) total score, ACE-R Fluency Score, ACE-R Visual-spatial score, Digit Span Test Forward, Verbal Fluency Test, Visual Search Test and Coloured Progressive Matrices, while it was positively correlated with Trail Making Test A. Fitting fully-adjusted models and independent of all covariates, the inverse association between CIRS-G score and Verbal Fluency Test was confirmed (*P* <.001), while no significant association was found with other cognitive tests. Noteworthy, we excluded that specific disease categories could have driven the association.

**Conclusions:**

The burden of multimorbidity is associated with impaired verbal phonemic fluency in individuals with normal cognition or MCI. Although further studies are required to confirm it, impaired verbal phonemic fluency may be an early sign of cognitive decline in older adults with multimorbidity, with potential implications for prevention strategies.

## Introduction

As the population ages, multimorbidity- which refers to the co-occurrence of multiple chronic conditions within a single individual [1] - is increasing presenting a significant global health challenge that affects individuals, healthcare, and society as a whole [2, 3]. Simultaneously, cognitive decline due to demographic changes is also on the rise and becoming a pressing global issue [4].

Age is widely recognized as the primary risk factor for most chronic diseases. While aging itself is not considered a disease, its underlying physiological changes increases susceptibility to illnesses and contribute to the onset of multimorbidity [5]. As individuals age, they accumulate cellular and molecular damage across multiple organs and systems, leading to significant changes in both structure and function. Once these changes reach a certain threshold of functional impairment, they become clinically evident as multiple chronic diseases [6]. Consequently, aging and multimorbidity are closely interconnected. Moreover, in older adults, multimorbidity is associated with a higher risk of adverse outcomes, including hospitalization [7]institutionalization [8]disability [9]and mortality [10, 11].

One of the most common aspects of aging is the decline in cognitive abilities. However, there is a significant variability among individuals in how quickly they experience this decline as they age, even in the absence of overt dementia. Early recognition of cognitive impairment is essential for timely intervention.

It is crucial to understand the link between the burden of multimorbidity and cognitive decline in older adults to develop preventive strategies that foster healthy aging.

Previous research suggests a bidirectional relationship between these two conditions [12]. Cognitive decline can increase the risk of developing multiple chronic conditions [13]while the presence of physical multimorbidity may elevate the risk of cognitive impairment and dementia. However, the impact of somatic multimorbidity on cognitive function in older adults remains a topic of debate.

It is particularly important to investigate how the burden of multiple chronic conditions affects cognitive function in older adults even without overt dementia. This investigation can help clarify the biological pathways that connect the deterioration of physical health with neurodegeneration and cerebrovascular pathology. Furthermore, identifying older adults with multimorbidity who are at higher risk of cognitive decline is essential to develop preventive measures aimed at enhancing cognitive health and supporting overall cognitive well-being among older adults facing multiple chronic conditions.

Notably, before a clinical diagnosis of dementia, many individuals experience a prolonged preclinical phase during which subtle neurodegenerative and cognitive changes may occur [14]. A recent research has shown that chronic diseases tend to accumulate more rapidly in individuals with prodromal dementia compared to those who are aging normally [15].

Also, there is growing evidence that having multiple chronic diseases increases the risk of developing dementia [16–23]especially when the onset of multimorbidity in at middle-ages rather than at old ages [17]. Moreover, a few population-based studies has investigated the relationship between multimorbidity and cognitive decline in older adults without dementia. In these studies, multimorbidity has primarily been defined as a simple count of diagnosed diseases, resulting in significant variability in the types and numbers of chronic conditions studied. Even with some exceptions [24]a greater number of multimorbidity and a faster accumulation of chronic diseases have been associated with accelerated cognitive decline [25–34]. Moreover, several studies have focused on specific clusters of chronic conditions [35–37]finding that cardiometabolic multimorbidity is particularly linked to the onset of dementia [38–41]. However, the association between morbidity burden indices [42]such as the Cumulative Illness Rating Scale-Geriatrics (CIRS-G), and cognitive function have not been exhaustively investigated in older adults with normal cognition or mild cognitive impairment (MCI).

Besides, it is important to note that real-world data are essential for capturing the complex interplay of multiple chronic conditions in older adults. Nevertheless, real-world evidence regarding the association between multimorbidity burden and cognitive decline is still limited.

This retrospective study utilized real-world data from electronic health records (EHRs) of older adults who were either cognitively intact or had mild cognitive impairment. These individuals were enrolled in the GERIatric COgnitive evaluation (GERICO), developed by the University of Perugia, Italy. This study aimed to investigate the relationship between the burden of multimorbidity, as measured by the Cumulative Illness Rating Scale-Geriatrics (CIRS-G), and cognitive impairment. Additionally, throughout an extensive battery of neuropsychological tests, this study sought to identify which cognitive domains were most significantly affected in older adults who had multimorbidity but did not exhibit overt dementia.

## Materials & methods

### Participants

The GeriCo (GERIatric COgnitive Evaluation, https://gericoev.eu) is a large Italian clinical-based study, promoted and developed by the Gerontology and Geriatrics Division of the University of Perugia, focused on cognitive impairment and dementia in old age subjects. The project consists of an electronic health record system for collecting clinical, biological, and neuroimaging data on older persons with cognitive and somatic problems. More details about the project have been previously published [43–44].

Participants were evaluated at the outpatient memory clinic for cognitive decline of the Centre for Studies on Cerebral Aging and Dementia of the University of Perugia, Italy. A total number of 1731 individuals was recruited between February 2016 and June 2023.

All participants recruited provided informed consent, and the study adhered to the Declaration of Helsinki and was approved by the Regional Ethical Committee (Prot. n. 8005/16/ON).

For the current secondary analysis, we included 898 participants with at least one visit with available complete information on the burden of multimorbidity and without diagnosis of overt dementia (specifically, individuals with Clinical Dementia Rating Scale equal to 0 (intact cognition) or 0.5 (mild cognitive impairment, MCI).

### Measurements

#### Multimorbidity

The burden of multimorbidity was operationalized by the CIRS-G, a well-validated and standardized clinical scale used as an indicator of global health status in older individuals [45]. The CIRS-G scale evaluates the clinical and functional severity of 14 anatomical categories of illnesses, including heart diseases, vascular diseases, hematopoietic disorders, respiratory diseases, eyes, ears, nose, throat, and larynx diseases, upper gastrointestinal disorders, lower gastrointestinal disorders, liver, pancreas, and biliary diseases, renal diseases, genitourinary diseases, musculoskeletal and skin diseases, neurologic diseases, endocrine and breast diseases, psychiatric illnesses. For each category, severity may range from 0 (no impairment) to 4 (extremely severe impairment). In our sample, the total CIRS-G score ranges from 0 to 25, and the tertile distribution was used to define the low, medium, and high burden of multimorbidity. The thresholds for tertiles of CIRS-G were 6 and 10, respectively for the 33rd and the 66th percentile.

#### Cognitive assessment

Cognitive function was assessed for each participant by trained psychologists using a comprehensive neuropsychological assessment. Participants underwent first-level examination using the screening test Addenbrooke’s Cognitive Examination-Revised (ACE-R) [46]. This is a brief cognitive battery scale assessing five subdomains: orientation-attention, memory, fluency, language, and visuospatial domains. ACE-R maximum score is 100, obtained by adding the above sub-scores, and also incorporating the Mini-Mental State Examination (MMSE) [47, 48] items and the Clock Drawing Test (CDT). Then, a second-level neuropsychological battery was administered to assess more in depth multiple cognitive domains, such as learning and memory, complex attention, executive functions, language, and perceptual–motor function, according to DSM 5. In particular, it includes: the Digit Span Forwards and Backward [49] tests for assessing short-term verbal memory and working memory respectively; the Rey Auditory Verbal Learning test immediate and delayed recall (RAVLT) [50, 51] and the logical memory test by Babcock [52] story to assess long-term verbal memory; the Corsi-span [53] for visuospatial short-term memory; the Trail Making Test A (TMT A) and Trail Making Test B (TMT B) [54] to assess attention and executive functions. Attention was evaluated using Attentional Matrices (Visual Search Test) [52]; the verbal phonemic and semantic fluency with the Letter (FAS) [51] and Categorial (FVC) [52] fluency Tests, respectively. The Token test [52] was used to assess auditory comprehension. The Raven’s test (Coloured Progressive Matrices- CPM47) [51] was used to assess fluid intelligence and logical reasoning. Finally, intelligence and executive function were evaluated by the verbal logical reasoning skills test [52].

Diagnosis of MCI considered both non-amnestic and multiple cognitive domain presentations with preserved independence in “essentially normal” functional activities. Then, criteria adopted for MCI included (1) change in cognition recognized by the affected individual or observers; (2) objective impairment in one or more cognitive domains; (3) independence in functional activities; and (4) absence of dementia.

#### Covariates

Age and education were assessed in years. Sex was included as a binary variable (1 = men; 2 = women). Functional status was assessed using the basic Activities of Daily Living Scale (ADL) and instrumental activities of daily living (IADL) scores. Specifically, ADL scores explore independence in six functions: bathing, personal hygiene, dressing, toilet hygiene, functional mobility, and self-feeding. A score of 6 indicates full function, 4 indicates moderate impairment, and 2 or less indicates severe functional impairment [55]. IADL scores evaluate independence in more complex functions, including using a telephone, shopping, meal preparation, housecleaning, laundry, transportation, managing finances and medications. A score of 8 indicates high function (fully independent), while a score of 0 indicates a severe impairment (fully dependent) in those skills [56]. The total number of current medications was also included as a covariate.

### Statistical analysis

Characteristics of our sample population were described as mean ± standard deviation (SDs), median, and interquartile range (IQR) or percentage. First of all, we explored the relationship between the burden of multimorbidity, operationalized as CIRS-G total score, and cognitive function using the full neuropsychological battery described above, adjusting for age, sex, and education. Afterward, for each cognitive test, which resulted, in preliminary analyses significantly correlated with multimorbidity, a multivariate linear regression model was performed to formally test the association between the CIRS-G total score and cognitive performance, independent of potential confounders, (age, sex, education, ADL and IADL score, and the total number of current medications). Finally, sensitivity analyses were performed using 14 different models, obtained by removing each time one of the 14 disease categories included in the CIRS-G scale. Results from sensitivity analyses were summarized as median and IQR of β coefficients and *p* values across the 14 regression models. All analyses were performed using the SAS statistical package, version 9.4 (SAS Institute Inc., Cary, NC) and R 4.3.1.

## Results

Overall 1532 participants presented at least one visit with complete information on the burden of multimorbidity and cognitive function. Of these, 898 (58.6%) individuals were not affected by dementia, specifically with Cumulative Dementia Rating Scale CDR = 0 (normal cognition) or with cognitively healthy or with CDR = 0.5 (MCI), and were included in the current analysis. Mean (± SD) age was 76.2 (± 8.3), min 41 max 98 years. Men were 345 (38.4%).

Characteristics of the sample population are summarized in Table 1 and reported by tertile distribution, of CIRS-G total score (low, medium, high burden of multimorbidity. Individuals with a higher burden of multimorbidity were older, had lower education, lower ADL scores, lower IADL scores, and a greater number of current medications, and were more likely to be cognitively mildly impaired (MCI).

**Table 1:** Participants’ ccharacteristics described by tertile distribution of the CIRS-G score across the entire sample, representing Multimorbidity burden classified as low, medium, or high

	Low burden of multimorbidity (low tertile of CIRS-G total Score)	Medium burden of multimorbidity (medium tertile of CIRS-G total Score)	High burden of multimorbidity (high tertile of CIRS-G total Score)
**N**	269	327	302
**Age**,** years**	72.3 (± 9.9)	76.9 (± 7.3)	78.9 (± 6.1)
**Sex (male)**	89 (33.1%)	137 (41.9%)	119 (39.4%)
**Education**,** years**	13 [5–13]	8 [5–13]	8 [5–13]
**ADL scores**	6 [5–6]	6 [5–6]	5 [5–6]
**IADL scores**	7 [5–8]	6 [4–8]	5 [3–7]
**Number of Medications**	3 [2–4]	4 [3–6]	7 [4–9]
**CIRS-G total score**	4 [3–5]	7 [6–8]	12 [11–15]
**Normal cognition** **(CDR = 0)**	147 (54.6%)	131 (40.1%)	109 (36.1%)
**Mild cognitive impairment** **(CDR = 0.5)**	122 (45.3%)	196 (59.9%)	193 (63.9%)
**MMSE**	29 [26–30]	27 [25–29]	27 [24–29]
**ACE-R** **Total score**	83 [68–93]	76 [65–87]	71 [63–82]
**ACE-R** **Attention/orientation**	17.5 [16–18]	17 [15–18]	17 [15–19]
**ACE-R** **Memory score**	18 [12–23]	15 [11–21]	14 [10–19]
**ACE – R** **Language score**	25 [22–26]	24 [21–25]	23 [20–25]
**ACE-R** **Fluency score**	9 [6–11]	8 [5–10]	7 [5–9]
**ACE-R visuospatial score**	15 [12–16]	14 [11–15]	13 [11–15]
**CDT**	5 [3–5]	4 [2–5]	4 [2–5]
**Digit span test forward**	5 [4–6]	5 [4–6]	5 [5–6]
**Digit span test backward**	3 [3–4]	3 [3–4]	3 [3–4]
**Rey Auditory Verbal Learning test (immediate)**	32 [23–44]	28 [21–35]	26 [20–31]
**Rey Auditory Verbal Learning test (delayed)**	6 [2–10]	4 [1–7]	4 [1–6]
**Babcock Story Logical memory test**	9 [4-12.5]	7.5 [4–11]	7.1 [3-10.4]
**Corsi block-tapping test**	4 [4–5]	4 [4–5]	4 [4–5]
**TMT-A**	51 [37–80]	65 [50–99]	75 [54–100]
**TMT-B**	118 [73–230]	164 [100–300]	180 [120–400]
**Verbal Phonemic Fluency Test**	29 [21–42]	25 [18–33]	22 [15-30.5]
**Verbal Semantic fluency Test**	16.25 [11.25-23]	13.5 [10.75-18]	13 [10-16.25]
**Visual search test**	47 [37–54]	40 [32–49]	38 [31–46]
**Raven’s Coloured Progressive Matrices (CPM47)**	27 [22–31]	25 [20–29]	23 [18–27]
**Token Test**	32 [30–34]	32 [29–34]	31 [29–33]
**Verbal logical reasoning skills test**	50 [41–56]	45 [35–54]	45 [36–52]

The association between the burden of multimorbidity, operationalized as CIRS-G total score, and multiple cognitive functions was first explored using Spearman’s correlations. After adjusting for age, sex, and education, we found that higher CIRS-G score was significantly and negatively correlated with ACE-R Total score, ACE-R Fluency Score, ACE-R Visual-Spatial score, Digit Span Test Forward, verbal phonemic fluency test, visual search test and Raven’s CPM47, while it was positively correlated with TMT-A (Table 2).

**Table 2:** Spearman correlations between CIRS-G total score and cognitive performance using a comprehensive battery of neuropsychological tests

		CIRS-G			
Cognitive function	*N*	Un-adjusted		Age, sex and education- adjusted	
		*r*	*P* value	*r*	*P* value
**MMSE**	898	-0.19	< 0.001	-0.05	0.099
**Addenbrook’s Cognitive Examination Revised Total score (ACE-R)**	873	-0.23	< 0.001	-0.07	**0.040**
**ACE-R Attention/Orientation domain**	873	-0.14	< 0.001	-0.02	0.562
**ACE-R** **Memory score**	873	-0.18	< 0.001	-0.03	0.382
**ACE-R** **Language score**	873	-0.18	< 0.001	-0.02	0.503
**ACE- R** **Fluency score**	873	-0.23	< 0.001	-0.11	**< 0.001**
**ACE-R** **Visuo-spatial score**	873	-0.20	< 0.001	-0.08	**0.011**
**Clock drawing Test (CDT)**	890	-0.09	0.004	-0.003	0.929
**Digit span test forward**	811	-0.16	<0.001	-0.09	**0.007**
**Digit span test backward**	808	-0.15	*P* <.001	-0.05	0.148
**Rey Auditory Verbal Learning test (immediate)**	761	-0.22	< 0.001	-0.04	0.174
**Rey Auditory Verbal Learning test (delayed)**	761	-0.19	< 0.001	-0.03	0.400
**Babcock Story Recall** **Logical memory Test**	757	-0.13	< 0.001	-0.19	0.594
**Corsi block-tapping test**	788	-0.14	< 0.001	-0.06	0.066
**Trail Making Test A**	777	0.26	< 0.001	0.08	**0.025**
**Trail Making Test B**	655	0.26	< 0.001	0.07	0.081
**Verbal Phonemic Fluency Test**	743	-0.26	< 0.001	-0.13	**< 0.001**
**Verbal Semantic fluency Test**	739	-0.21	< 0.001	-0.04	0.224
**Visual search test**	791	-0.25	< 0.001	-0.11	**0.002**
**Raven’s Coloured Progressive Matrices (CPM47)**	764	-0.24	< 0.001	-0.12	**< 0.001**
**Token Test**	690	-0.12	0.001	-0.03	0.454
**Verbal logical reasoning skills test**	744	-0.17	< 0.001	-0.07	0.071

Then, we conducted a multivariate linear regression model to confirm these associations. Independently of age, sex, education, ADL and IADL scores, and the total number of current medications, a higher CIRS-G score was significantly associated with a lower verbal phonemic fluency test (*P* <.001). ACE-R Fluency Score showed a similar association, although it did not reach statistical significance (*P* =.087). No significant association was found between CIRS-G and the other cognitive tests (Table 3).

**Table 3:** Multivariate linear regressions testing the association between CIRS-G total score and cognitive performance

	Cognitive Test		Sensitivity Analyses	
	Addenbrook ’s Cognitive Examination Revised (ACE-*R*)			
	β (SE)	*P* value		
**CIRS_G**	-0.03 (0.12)	0.800		
	***ACE-R Fluency Score***			
	**β (SE)**	**P value**		
**CIRS_G**	-0.05 (0.03)	**0.087**		
	***ACE-R Visuo-spatial Score***			
	**β (SE)**	**P value**		
**CIRS_G**	-0.01 (0.02)	0.730		
	***Digit Span Test Forward***			
	**β (SE)**	**P value**		
**CIRS_G**	-0.01 (0.01)	0.234		
	***Trail Making Test A***			
	**β (SE)**	**P value**		
**CIRS_G**	-1.06 (0.67)	0.115		
	***Verbal Phonemic Fluency Test***			
	**β (SE)**	**P value**	**Β median (IQR)**	**P value median (IQR)**
**CIRS_G**	-0.46 (0.14)	**< 0.001** ^*, #^	**-0.47** **(-0.48**, **-0.45)**	**0.001** **(< 0.001**,** 0.002)**
	***Visual Search Test***			
	**β (SE)**	**P value**		
**CIRS_G**	0.05 (0.12)	0.661		
	***Colored Progressive Matrices***			
	**β (SE)**	**P value**		
**CIRS_G**	-0.06 (0.07)	0.395		

Legend: covariates included in the analysis are age, sex, education, ADL and IADL scores, and total number of current medications

^*^P <.05 at Bonferroni correction; ^#^*P* <.05 at Benjamini–Hochberg procedure

**Fig. 1:**
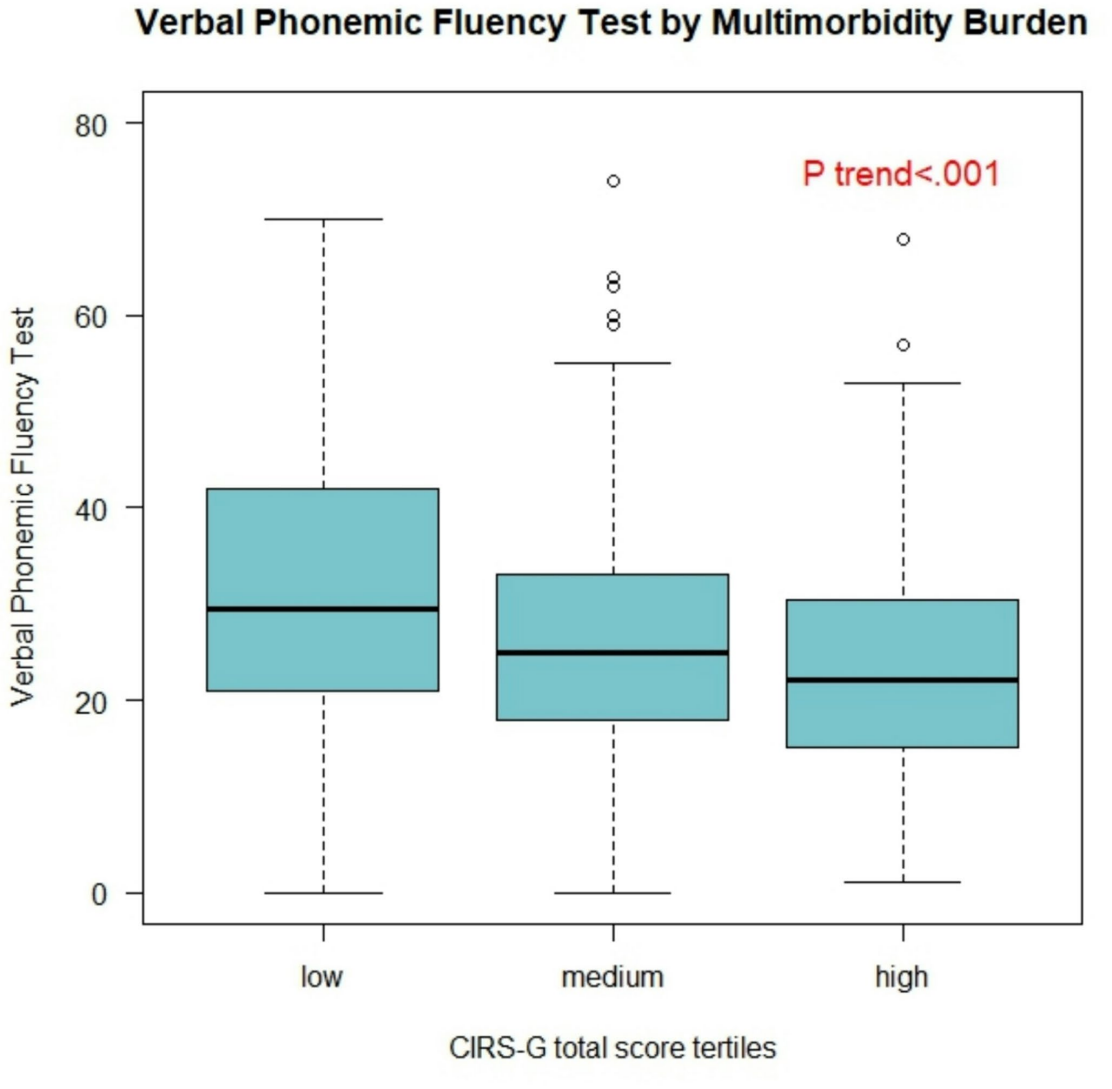
Median values of Verbal Phonemic Fluency Test according to different tertiles of CIRS-G total score, corresponding to the low, medium, and high burden of multimorbidity

Figure 1 represents median values of verbal phonemic fluency test, according to different tertiles of the CIRS-G total score, corresponding to the low, medium, and high burden of multimorbidity.

Independent of covariates, individuals with a high burden of multimorbidity—defined as being in the highest tertile of CIRS-G in our sample—showed a significant reduction of 3.44 points in the verbal phonemic fluency test compared to those in the lowest tertile of CIRS-G.

Moreover, the result was substantially retained after conducting multiple comparison corrections using Bonferroni and Benjamini–Hochberg procedures (Table 3).

Finally, sensitivity analyses were performed in order to exclude that one specific disease category may drive the association with verbal phonemic fluency. In particular, we fitted 14 different regression models, each one using a new version of the CIRS-G scale recalculated by eliminating one by one each of the 14 categories included in the original CIRS-G. The results across the 14 regression models, summarized in Table 3 as median and IQR of β coefficients and *p* values across the 14 regression models, were consistent with the original analysis, suggesting that association were not driven by a specific disease category.

## Discussion

The present study investigated the relationship between the burden of multimorbidity, estimated by using the CIRS-G scale, and cognitive function in individuals with normal cognition or mild cognitive impairment. The results showed a significant negative association between the burden of multimorbidity and verbal phonemic fluency, even after controlling for potential confounders such as age, sex, education, activities of daily living (ADL) and instrumental activities of daily living (IADL) scores, as well as the number of current medications. Additionally, we ruled out the possibility that specific disease categories were responsible for this association.

Our results significantly enhance the existing evidence supporting the link between multimorbidity and cognitive performance. Consistent with our analysis, previous studies have shown that multimorbidity is a risk factor for cognitive impairment and dementia [16–23]and accelerates cognitive decline in older adults [25–34]. However, the mechanisms underlying this association are still not fully understood. One plausible hypothesis is that multimorbidity could have a negative impact on the accumulation of subclinical damage in the brain, accelerating the transition from normal cognitive function to mild cognitive impairment [25]. Also, inflammation increases the risk of dementia in individuals with multimorbidity [16].

Both preclinical and epidemiological studies support the idea that multimorbidity and aging share common biological underpinnings, including chronic inflammation, oxidative stress, mitochondrial dysfunction, cellular senescence, and epigenetic alterations [57, 58]. Such biological mechanisms, often referred to as “Hallmarks of Aging”, contribute to a progressive loss of resilience and damage accumulation across multiple organs and systems, leading to accelerated aging and the development of multiple chronic diseases [59]. In the brain, the same biological mechanisms drive the progressive loss of cognitive function with aging [60]. Therefore, multimorbidity could increase the risk of cognitive decline by accelerating damage accumulation within the brain. Supporting this hypothesis, a recent study showed that individuals with multimorbidity exhibit accelerated structural brain changes, including both neurodegeneration and vascular pathology, compared to those without multimorbidity [61]. Based on this concept, older adults with high burden of multimorbidity but preserved cognitive functions might be key candidates for interventions aimed at mitigating the hallmarks of aging, especially those related brain aging and neurodegeneration [5, 62].

An alternative hypothesis suggests that factors associated with multimorbidity—such as loss of physical function and performance, treatment burdens, polypharmacy, declining social interactions, and psychological consequences—may predispose individuals to accelerated cognitive decline [63, 64]. It remains unclear whether multimorbidity directly contributes to cognitive impairment or simply serves as an indicator of imminent cognitive deterioration. Further investigations are necessary to fully understand the complex relationship between physical and cognitive health.

Our findings indicate that the burden of multimorbidity has a specific impact on verbal phonemic fluency. After controlling for potential confounders, we found no significant association with other cognitive domains. This result aligns well with a previous study involving older participants without dementia from the Baltimore Longitudinal Study of Aging (BLSA), which showed that a faster accumulation of chronic diseases was significantly and specifically associated with a more rapid decline in performance on the Category and Letter Fluency Tests [26]. The specific mechanisms linking multimorbidity to verbal fluency remain largely unknown. Previous research has indicated that multimorbidity is associated with reduced volume of specific brain regions, such as the hippocampus, posterior cingulate cortex, supramarginal cortex, and right precuneus cortex [61, 65, 66]. The hippocampus is known to particularly support semantic verbal fluency [67]while the posterior cingulate cortex and supramarginal cortex may be involved in verbal phonemic fluency [68, 69]. Furthermore, multimorbidity has been correlated with lower brain activity in the frontal and temporal regions, which are crucial for verbal fluency [70].

Verbal fluency is known to be impaired in individuals with mild cognitive impairment (MCI) compared to those who are cognitively healthy. Deficits in verbal fluency may emerge several years before the diagnosis of MCI or dementia, making it a valuable early preclinical screening tool for impending cognitive decline [71, 72].

Additionally, higher cognitive reserves have been associated with better performance in verbal phonemic fluency among healthy older adults [73].

Thus, we propose that the disruptions in homeostasis across multiple organs and systems due to the accumulation of multimorbidity may manifest in the brain level as a progressive loss of cognitive reserves. This loss may be evident early on as impairments in phonemic fluency, even in individuals with normal cognition or mild cognitive impairment.

The benefits of early detection of cognitive decline are undeniable. Recognizing cognitive issues early enables timely intervention, effective symptom management, psychosocial support, and improved quality of life for both patients and their families. Unfortunately, mild cognitive impairment is often underdiagnosed in older adults with multiple chronic conditions. The presence of overlapping health issues can mask cognitive symptoms, leading to delayed or missed diagnoses. Additionally, early signs of cognitive impairment may be mistaken for normal age-related decline or attributed to medication side effects. Therefore, regular cognitive assessments for individuals with multiple chronic conditions are essential for the early detection of cognitive impairment.

Our study suggested that decreased performance in verbal phonemic fluency might be an early indicator of reduced cognitive reserves in individuals with multiple chronic conditions. Verbal phonemic fluency was evaluated using the Letter Fluency Test (FAS), a commonly used tool in neuropsychological assessments for older outpatients. This test helps identify deficits and monitor changes in cognitive functions, particularly in language skills, executive functions, and working memory. Patients are asked to name as many words as possible within a 60-second time limit for each of the letters F, A, and S, making the entire test take about 3 min. Because of its simplicity, cost-effectiveness, and quick administration, we believe that physicians should incorporate regular cognitive assessments into the clinical care of older adults with multiple chronic conditions.

The primary strength of the study lies in the thorough cognitive and clinical assessment that participants received upon their admission at the outpatient memory clinic. Specifically, participants underwent extensive neuropsychological evaluations, as well as complete clinical assessments to identify multi-organ diseases, geriatric syndromes, and issues related to polypharmacy. Furthermore, multimorbidity was assessed not merely by counting diseases, but through an index that evaluates the medical burden associated with multimorbidity.

However, several limitations need to be addressed. First, further research involving larger and more diverse populations is necessary to validate our findings and confirm their generalizability. Second, we could not definitively establish a causal relationship between the burden of multimorbidity and declines in cognitive impairment and verbal phonemic fluency due to the cross-sectional design of our study. It’s important to consider the possibility of reverse causality. Therefore, additional longitudinal studies are needed to clarify the causal relationship between multimorbidity and cognitive impairment fully. Furthermore, multimorbidity is closely linked to polypharmacy, meaning that as a patient’s health declines, they tend to take more medications. Although our sample population did not show a significant relationship between the total number of current medications and verbal fluency, we cannot dismiss the possibility that specific medications could influence verbal fluency performance. Notably, previous research has indicated that the use of potentially inappropriate medications – identified using the STOPP criteria - was associated with lower verbal fluency in older adults who have mild cognitive impairment (MCI) or mild dementia [74]. Further studies are needed to fully investigate whether certain drugs or combinations of drugs may affect verbal fluency in the geriatric population with multiple chronic conditions.

## Conclusions and implications

Using real-world Electronic Health Records data, we demonstrated that the burden of multimorbidity is associated with impaired verbal phonemic fluency in individuals without dementia. While further research is needed to fully understand the underlying mechanisms and establish a causal pathway, one plausible explanation is that the accumulation of multisystem damage associated with aging—typical of multimorbidity—may lead to a gradual depletion of cognitive reserves at the brain level. This decline in cognitive reserves may manifest as a deficit in verbal phonemic fluency, particularly in the early stages for individuals with normal cognition or mild cognitive impairment (MCI).
